# Redefining medical education: harnessing the power of patient feedback

**DOI:** 10.3389/fmed.2024.1453262

**Published:** 2024-10-18

**Authors:** Waseem Jerjes, Daniel Harding

**Affiliations:** ^1^Research and Development Unit, Hammersmith and Fulham Primary Care Network, London, United Kingdom; ^2^Faculty of Medicine, Imperial College London, London, United Kingdom

**Keywords:** patient feedback, medical training and education, health education paradigms, communication skills, medical practice

## Introduction

Health education paradigms are changing and now increasingly emphasize the importance of patient-centered care. This change has not occurred as a trend but is a basic tenet in training and preparing healthcare professionals to serve patient populations with complex needs. Central to such a change is the integration of patient feedback into the process of clinical education: a practice that centralizes the patient's perspective at the very core of the development of a clinician (1). Such feedback in medical training is not just an adjunct to traditional education models; rather, it actively enriches by providing real-time insights into the utility of medical communication, empathy, and patient autonomy. This view point will argue why we believe patient feedback must be better valued and utilized in clinical education. This is not only to enhance the communication skills that shape patient interaction but moreover to instill a patient-centric philosophy, vital to developing our future clinicians into responsive, empathetic, socially-conscientious clinicians. For this reason, we assess the value of patient feedback in clinical education by highlighting its importance in an educational context where healthcare professionals of the future can easily manage the complicated interpersonal dimensions of medical practice. Our focus will be on undergraduate medical education, but we believe similar ideas are applicable to postgraduate specialist medical education. The debate that will ensue herein will look at the historical background of this integration, the multifaceted benefits of patient feedback, and then more practically, the challenges and potential solutions to its implementation.

### Historical overview and current shifts

Traditionally, clinical education has been driven by a clinician-centric model. Following the impact of the Flexner report, for over a century medical education has emphasized university-based didactic teaching of biomedical sciences, participation in medical research, and bedside teaching of technical skills by specialist physicians (1, 2). This profoundly entrenched approach formed part of broader historical practices that privileged the medical professional's expertise—traditionally at the expense of broader patient engagement. Further, the overemphasis in medical education on “make or break” qualification examinations has arguably led to neglect around development of the holistic skills required to be a well-rounded clinician (3). While this model is effective in developing technical competence, it has often proved to be limited by failure to recognize the most essential participant in the educational dialogue—the patient, and thus learning has been defined as clinical interaction and not holistic patient care. Further, such technical skills may well lose relevance over the course of a clinician's career as new technology emerges and the landscape of healthcare changes.

Over the last decade, however, there has been a significant shift toward an expanded view of education that actively includes the patient (4). This changing nature is, in part, a growing appreciation for the powerful depth of insights that patients bring to the healthcare experience—truly priceless insights to help train future empathetic, responsive, and patient-centered professionals. This integration of patient perspectives into an educational frame represents a gargantuan cultural change in medical education: acknowledgment that patients are active and necessary contributors to the learning process. Acceptance of this change further aligns educational practice with emerging expectations from patients, and indeed from the medical profession itself, while at the same time signaling a move to more dynamic medical education (5).

Certainly some alternative models have emerged that challenge the historically-dominant, Western style of medical education. For instance, the Mayo Clinic model has emphasized developing professionalism and regard for patient welfare in students, something integrated at all levels of education, including assessment (6). Additionally, several distinct schools, such as Northern Ontario (Canada) and Ateneo de Zamboanga University (the Philippines) Schools of Medicine, have developed novel programs which centralize education around community-engagement in rural underserved areas, and seek to develop socially conscientious practitioners (7, 8).

### The value of patient feedback

The pillar of the transformational approach to clinical education is on utility brought out in terms of patient feedback, given from experience and mostly overlooked in the conventional models of teaching. Therefore, this kind of feedback is crucial since it reflects their direct experiences and perceptions about care (9). Types of feedback commonly gathered include: how well one communicated, demonstrated empathy and listening, empowered the patient and respected their autonomy, or educated the patient about matters related to health. The type of feedback can range from simple quantitative scoring to detailed qualitative feedback and instruction.

Integrating such feedback highlights the often-subtle interpersonal dynamics in patient interaction that can significantly influence patient satisfaction and treatment adherence (10). For example, tailored patient feedback can identify weaknesses, and motivate students toward developing more considerate, and therefore more effective, practice. Educator exposure to such feedback, may also help positively shift educational culture and methods toward considering the patient perspective. Indeed, from a course design point of view, the use of patient feedback for the development of training material could be used as a focus for the enhancement of more straightforward, warmer communication skills. Additionally, such educational methods should help facilitate patients sharing their lived experience and expertise about their own conditions, greatly contextualizing and broadening student understanding of these conditions (11).

Furthermore, personal autonomy and empathy feedback can guide the understanding and promotion of patient trust and confidence—vital in any clinical relationship. Through a systematic infusion of these findings into the curriculum, these educators would thus be nurturing a generation of technically competent healthcare professionals who are also brought up to be deeply sensitized toward the social, emotional, and psychological facets of patient care. It further helps in more patient-centric, empathetic healthcare, but ultimately also elevates the standards of care (12). Through ensuring high quality communication, benefits such as active patient participation, better self-care, and improved treatment adherence can be achieved. Closer and fuller collaboration between clinician and patient through such refined communication skills, has also been shown to enhance chronic disease measures and functional status (13). Vitally, this also ensures the care delivered is of relevance to meeting a patient's priorities, having fully explored and understood them beforehand. Integration of patient feedback, can also enhance awareness and education about local communities, so long as patients are recruited from a broad range of cultural, ethnic, and socioeconomic backgrounds. This could be promoted through engaging diverse community stakeholders. Such an approach could expose students early on in their education to cultures differing from their own background, with an aim to enhancing social conscientiousness and cultural competence.

### Methods for gathering patient feedback

Systematic collection of patient feedback needs to be done with care to ensure processes respect the confidentiality and sensitivity of the patient-clinician relationship (14). Different methodologies have been used to gather such invaluable data, ensuring process integrity and the comfort of all parties involved. A usual technique is the implementation of questionnaires with their patients remaining unidentified or, for instance, structured interviews through independent third parties—educational supervisors. Such instruments seek to investigate somewhat tangible aspects of clinical communication, such as communication skills, empathy, and the understanding of the patient, thus providing an explicit source of valuable information while keeping the patient anonymous.

Moreover, the presence of patient representatives in educational review committees adds huge depth and pertinence to the process of giving feedback (15). First-hand patient views are highly valuable to the development of the curricula and academic assessments, thus making sure that educational strategies closely follow the needs and expectations of the patients. This not only enriches the educational content but also vitally includes patients in shaping the training of healthcare professionals who will serve future generations. Innovative models of medical education have even successfully embedded patient-engagement heavily into medical school design and student admissions to ensure that local communities are best served. For example, Northern Ontario School of Medicine regularly includes community members in admission interview panels, consultations on curriculum design and development, and in facilitating community placements (7).

Both methods underlie the commitment to a more inclusive and responsive medical education system, where patient experiences are acknowledged and integral in shaping the educational paradigms (16). This will then bring about insightful knowledge and move medical education toward the development of genuinely patient-centered healthcare professionals. Table 1 shows the path along which educational institutions should find ways of embedding patient input effectively in their curricula.

**Table 1:** Structured plan for integrating patient feedback into clinical education.

**Stage**	**Description**	**Methods**	**Expected outcomes**
Preparation	Educate faculty and students about the value of patient feedback	Workshops, seminars, online modules	Improved understanding and acceptance of patient feedback processes
Recruitment	Ensure patients from diverse backgrounds, and reflecting local communities, are recruited	Proactive patient recruitment, community stakeholder engagement	More socially-accountable education and enhanced cultural and social awareness of learner
Collection	Systematic gathering of feedback through various methods	Surveys, interviews, focus groups	Diverse and comprehensive data on student performance and patient satisfaction
Analysis	Process feedback to extract actionable insights	Data analysis software, expert review panels	Identification of key areas for improvement and strengths
Implementation	Incorporate feedback into the curriculum and teaching methods	Revised teaching plans, case studies, simulation activities	Enhanced curriculum that addresses real-world patient needs and improves student empathy and communication skills
Evaluation	Assess the impact of feedback integration on student performance and patient care quality	Follow-up surveys, performance assessments, patient care outcomes	Tangible improvements in educational and healthcare delivery outcomes

### Integrating feedback into teaching practices

Patient involvement in teaching strategies is a relatively new technique, and its implementation might significantly impact how to train the generation of health professionals (17, 18). One new style is case-based learning, that includes stories and feedback from actual patients. This approach allows students to be fully immersed in patient experiences, which is meant to bring about a more intuitive comprehension of the principles of patient-centered care. When patients share their lived experiences, it brings practical reality to students so that they value the complexity of patient interactions and the importance of empathy, communication, and ethical practice.

Simulated patient encounters are another fruitful way: in this process, after being briefed on scenarios, actors give specific feedback based on their experience as a simulated patient (19, 20). This way, it becomes a safe learning environment for the students, who can develop and practice clinical and interpersonal skills without causing harm or distress to actual patients. Following these simulations, debriefing is critical in providing the opportunity for reflective learning, allowing students to receive immediate and constructive feedback to enhance both their technical skills and communication style and their ability to mentalize and respond to patient emotions and needs.

These are teaching practices meant to develop not only clinical competencies but also a professional attitude—patient perspective is considered an integral constituent of the development of professionalism (21, 22). Embedding these methods within the curriculum allows the medical educator to create a more dynamic learning environment, therefore enabling the learners to be more reflective and responsive ultimately resulting in competent health practitioners.

## Discussion

The integration of patient feedback into medical curricula is a strategic move that significantly realigns educational content with the principles of patient-centered care (1, 4, 23). This feedback is necessary for forming personalized learning objectives; not to mention, refining assessment criteria makes sure that the curriculum teaches not just medical expertise but also broader holistic skills, with efficient communication with patients.

In this light, an educator can develop a curriculum enriched with patient stories and feedback to emulate the kind of clinical practice where communication, empathetic responses, and patient autonomy are stressed as vital to the successful application of clinical skills; which are ultimately more relevant and acceptable to the patient (24, 25). Such stories provide rich context scenarios that challenge the student to think beyond the diagnosis to get the experience, fears, and psychological wellbeing of the patient. This method promotes a holistic view of medicine that values the patient's perspective as a critical ingredient in appropriate care.

In addition, the development of assessment tools that measure students' ability to meet these integrated objectives is directly influenced by patient feedback (26, 27). Classic assessments are relatively narrow and focus on technical skills and knowledge. With redefined criteria, due to patient feedback, the assessments can be more comprehensive regarding whether a student can effectively interact with patients, thus covering more competencies, such as interpersonal skills and ethical considerations, and at the same time being clear as to those that need targeting for personal development goals. That feedback, incorporated into curriculum development for improvement, would assure more effective training for healthcare professionals and better response to what communities await from their service. Alignment of this nature is essential in driving a healthcare system that is genuinely responsive and sensitive to the patient's experience in fostering good care outcomes, as well as helping us strive toward health equity and inclusivity goals amongst underserved communities.

### Broader implications for healthcare culture

Incorporating patient feedback into clinical education is far more than a pedagogical shift; it represents seismic change within healthcare cultures toward openness, inclusivity, and genuinely patient-centered systems (1, 12). This process in medical education will help us better respond to the broader social demand for the establishment of a medical practice that should be not only effective on a clinical level, but also compassionate and carefully crafted around patient's individual needs and preferences.

Educating health professionals to integrate patient views in learning strategies embraces a culture of respecting patient experience and views (28). It forms a cultural change way beyond the medical schools and trickles down through the health delivery ecosystem with an eventual betterment of patient care quality and patient outcomes. Such practice becomes normalized and improves the health environment when the principles of empathy, respect, and autonomy toward patients are concerned.

Most importantly, this long-term change bears profound benefits. It will improve patient satisfaction and engagement because new generations of healthcare providers, who were appropriately trained in the complexities of patient interactions, will be enabled (29). This offers great potential for achieving patient adherence to treatment plans and preventive measures, in the end, enhancing health outcomes and lowering the costs of care. Furthermore, a culture that actively pursues and values the input of patients could become inherently self-improving, adapting constantly to the changing needs and expectations of the population it serves.

Therefore, embedding patient feedback into clinical education not only enriches the development of health professionals but also significantly contributes to the evolution of a healthcare system focused on the values for whom it is created.

### Challenges and considerations

Patient feedback on clinical education has much potential, yet it is not without its challenges or the possibility of being problematic. Caution will be needed in handling these barriers to ensure that the potentials of this educational method are realized and sustained.

One primary concern is if negative feedback might impact student confidence, particularly in the initial stages of training. While constructive criticism has its place in professional development, repeated or intense negativity might risk reducing self-esteem and impair effective learning (23, 30). As such, medical educators should promote a safe, encouraging learning environment, where students are encouraged to reflect and grow, but not in a way that takes away from them.

Major logistical problems also exist. “Taking feedback from patients, analysis of it and putting it into systems which are already overburdened in terms of clinical and educational activities is not always easy.” This requires a great deal of planning and provision of resources to ensure that the processes are well organized and effectively conducted so the feedback is also relevant and timely. This might sometimes require developing new administrative protocols or setting up technological solutions to streamline the feedback processes (1, 12, 26, 31).

Besides, it is essential that the fairness and representativeness of the feedback collected is of utmost importance (7, 20). It will be essential to gather feedback from as diversified a patient population as possible to not introduce bias that may distort educational content and results. This calls for intentional engagement with the highest levels of patient diversity and the health conditions they present so that any feedback resulting from the process can be applied in an all-inclusive manner and utilized across the patient's experience. Essential here is ensuring that those educational practices lessen health inequities rather than deepen them.

Overcoming these challenges requires a dynamic, thoughtful approach with continuous evaluation and modification of strategies to incorporate patient feedback into medical education effectively.

## Conclusion

It, therefore, underscores the need to turn around a paradigm regarding patient feedback in clinical learning to be within an approach that is more inclusive and empathic through which healthcare professionals can be trained. By and large, it would have been noticed throughout the discussion that patient feedback not only enriches the educational process but also sharpens future clinicians' interpersonal skills and the underpinning attitudes of care, hence improving patient care.

Medical educators, therefore, have the formidable charge of catalyzing such change. Incorporating patient feedback into the curriculum will create professionals in health education who are as technically competent as they are sensitized to the patient's wishes and experiences to whom they will deliver their services. This is the only way that a caring healthcare environment—one that empathizes with and values patients' experiences—will be realized.

Patient feedback, therefore, offers benefits not just to individual patient encounters but also to general healthcare outcomes. In essence, the medical community needs to adopt and spread patient-centered educational practices to ensure that changes in societal expectations are met.
